# Supplementary material for: Scalable recombinase-based gene expression cascades
Source: Nat Commun. 2021 May 11;12:2711. doi: 10.1038/s41467-021-22978-4 (PMC8113245; doi:10.1038/s41467-021-22978-4)
Supplement: Supplementary file 1 — Supplementary Information [file 41467_2021_22978_MOESM1_ESM.pdf]

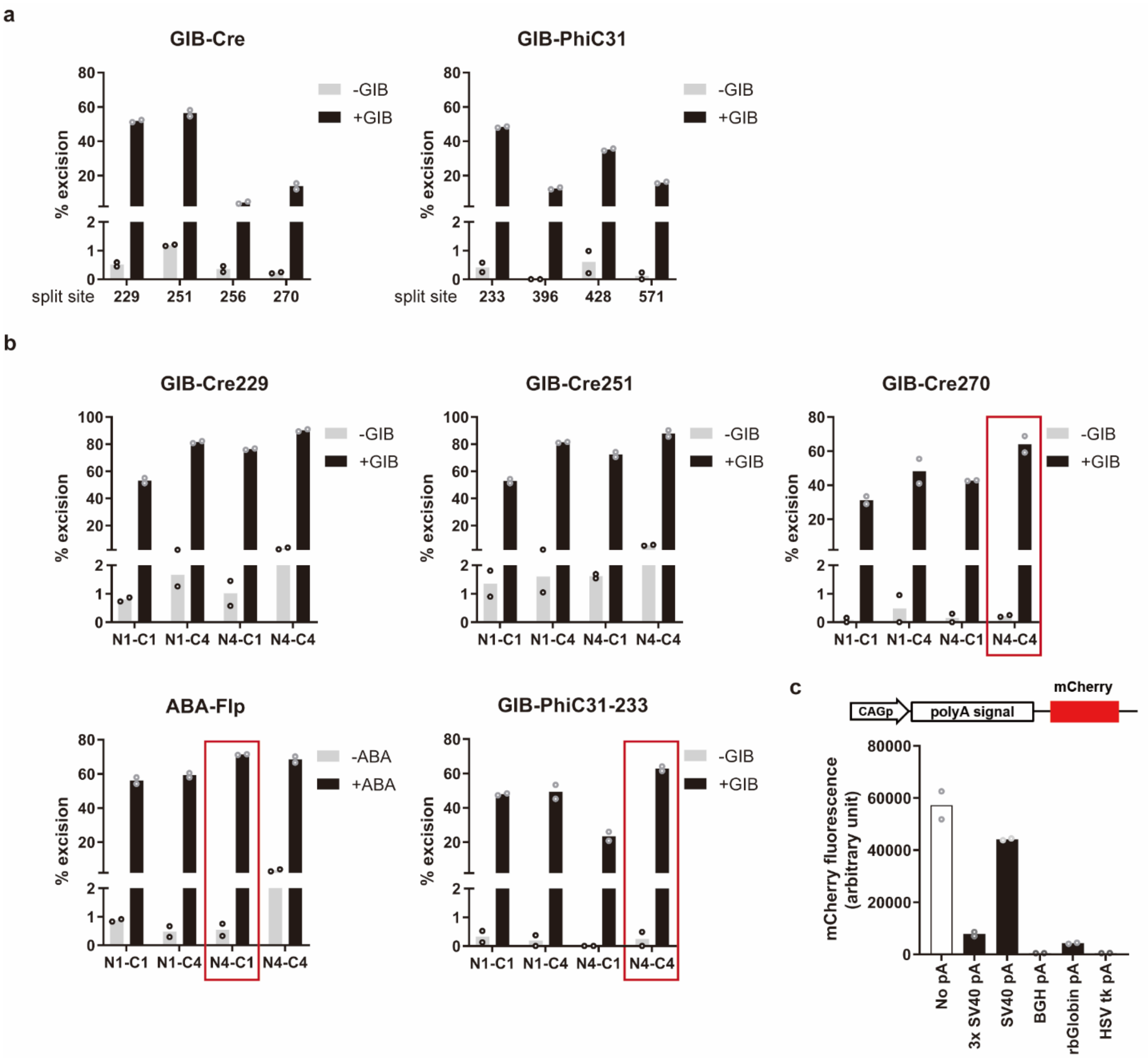

Supplementary Fig. 1. Optimization of unit GPCs. a-b) % BFP positive cells were calculated as % self-excision of GPCs with the experimental designs described in Fig. 1b. Excision frequency was calculated as %BFP+/%(BFP+ or GFP+) as was done for Fig. 1d. a, Determination of ideal recombinase split sites for minimal leakage in the absence of ligand and efficient ligand-induced self-excision of GPCs (n=2, values indicate mean). Split recombinases with site x involved N terminal fragment of amino acids 1-x, and C terminal fragment of amino acids x+1 to the end of the recombinase polypeptide. b, Determination of optimal number of nuclear localization signals (NLS) fused to each fragment of split recombinases for best activity in the presence of ligands (n=2, values indicate mean). Nx-Cy indicates that the N-terminal fragment of recombinase was fused with x NLS(s), and the C terminal fragment was fused with y NLS(s). Red rectangles indicate the optimized split-recombinases used throughout this study. c, Comparison of different polyadenylation signals for efficient transcription termination (n=2, values indicate mean). Source data are provided as a source data file.

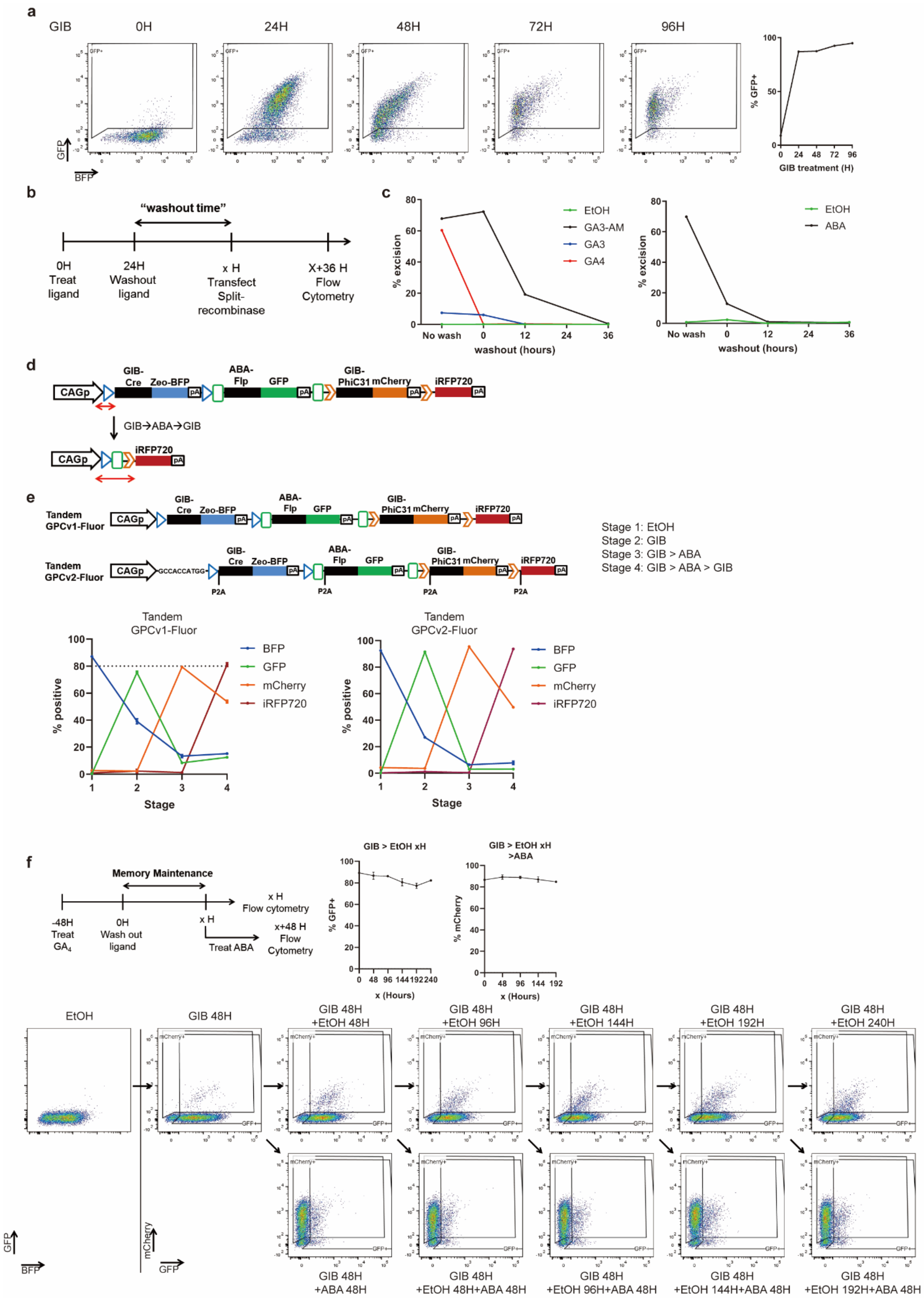
a
GIB
0H
24H
48H
72H
96H
GFP+
GFP
BFP
% GFP+
GIB treatment (H)
b
"washout time"
0H Treat ligand
24H Washout ligand
x H Transfect Split-recombinase
X+36 H Flow Cytometry
c
% excision
EtOH
GA3-AM
GA3
GA4
ABA
No wash
washout (hours)
d
CAGp
GIB-Cre
Zeo-BFP
ABA-Flp
GFP
GIB-PhiC31
mCherry
iRFP720
pA
GIB→ABA→GIB
e
Tandem GPCv1-Fluor
Tandem GPCv2-Fluor
GCCACCATGG
P2A
Stage 1: EtOH
Stage 2: GIB
Stage 3: GIB > ABA
Stage 4: GIB > ABA > GIB
% positive
BFP
GFP
mCherry
iRFP720
Stage
f
Memory Maintenance
-48H Treat GA4
0H Wash out ligand
x H
x H Flow cytometry
Treat ABA
x+48 H Flow Cytometry
GIB > EtOH xH
GIB > EtOH xH >ABA
% GFP+
% mCherry
x (Hours)
EtOH
GIB 48H
GIB 48H +EtOH 48H
GIB 48H +EtOH 96H
GIB 48H +EtOH 144H
GIB 48H +EtOH 192H
GIB 48H +EtOH 240H
mCherry+
GFP+
GIB 48H +ABA 48H
GIB 48H +EtOH 48H+ABA 48H
GIB 48H +EtOH 96H+ABA 48H
GIB 48H +EtOH 144H+ABA 48H
GIB 48H +EtOH 192H+ABA 48H
mCherry
GFP
BFP

Supplementary Fig. 2. Optimization of tandem GPC circuit. a, Kinetics of recombinase-induced excision after ligand treatment. Tandem GPCv2-Fluor was treated with GA_4 for indicated durations. Excision was monitored as % GFP positive cells. (Left) Representative flow cytometry plots, (Right) Quantification of the % GFP positive cells (n=2, values indicate mean). b-c) Kinetics of CID ligand decay. b, Cells were pretreated with various versions of gibberellin or abscisic acid. Gibberellin treated cells were transfected with plasmid encoding GIB-Cre GPC (used in fig. 1d) at indicated time points after ligand washout. ABA treated cells were transfected with plasmid encoding ABA-Flp GPC (used in fig. 1d) at indicated time points after ligand washout. c, Quantification of the % excised cells with GPCs delivered after various washout time (n=2, values indicate mean). d, Lengthening of "scar" 5'-UTR sequences after each excision. The "scar" sequence is indicated with the red arrow. e, Improved "tandem GPCv2" circuit design to prevent 5'-UTR lengthening. Line graphs below show the fraction of cells expressing indicated payload gene after each indicated sequence of ligand treatment. f, Memory maintenance by the gene expression cascade. (Top left) Experimental scheme. (Top right) Quantification of mean GFP level and fraction of GFP positive cells, and mean mCherry level after 48 hours of ABA treatment with fraction of mCherry positive cells (n=3, mean± s.d.) (Bottom) Representative flow cytometry plots. Source data are provided as a source data file.

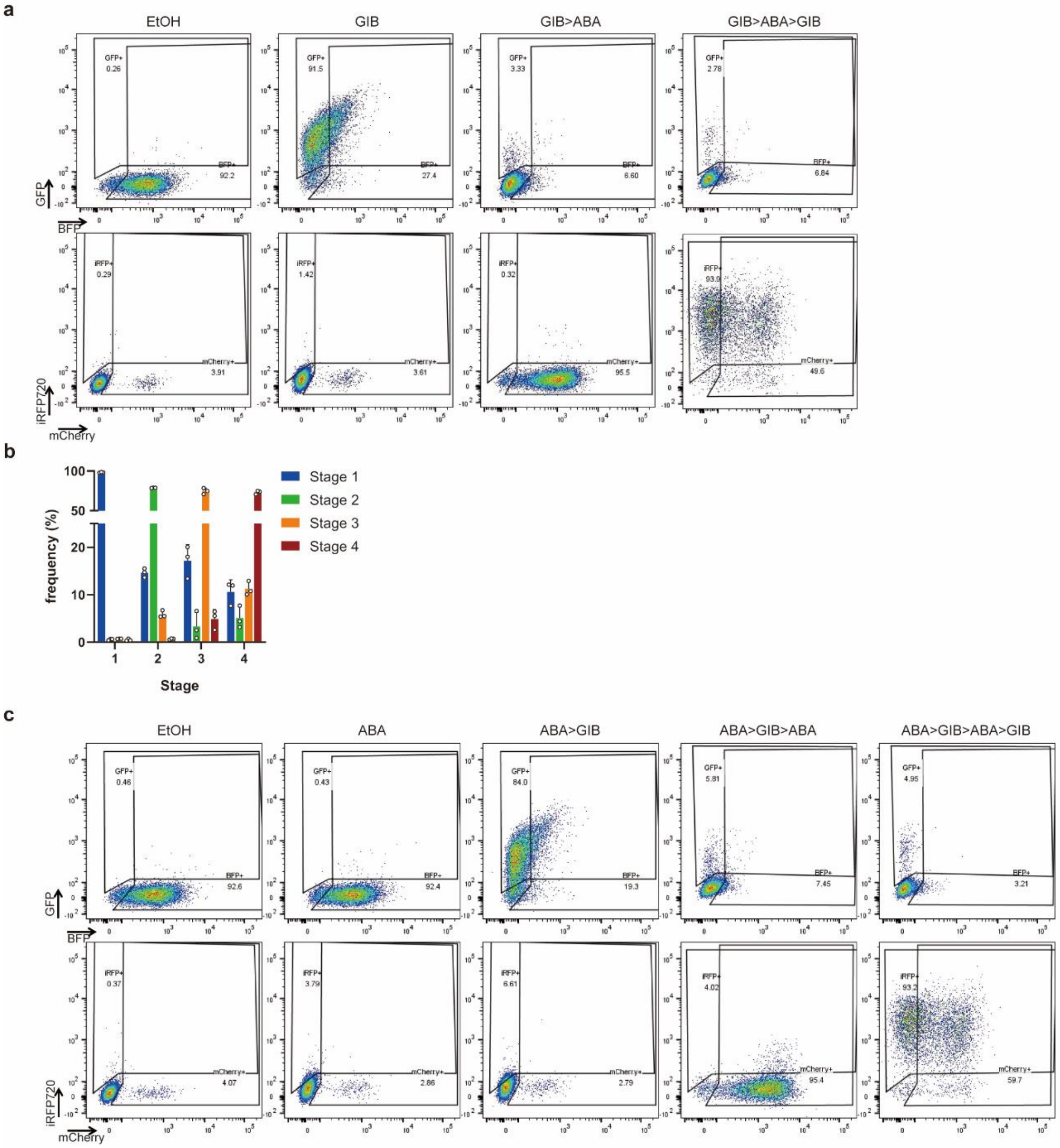

Supplementary Fig. 3. Tandem GPCv2-Fluor enables robust, sequential expression of fluorescent proteins. a. Flow cytometry plots for all relevant fluorescence channels for the data shown in Figure 2b-c. b. NGS analysis of the PCR amplicon consisting of scar sequence from cells used in Figure 2b-c. Each bar indicates the frequency of the detected scar sequence that corresponds to each stage in the cascade at the actual stage within the cascade, indicated below (n=3, mean ± s.d.). c. Flow cytometry plots for all relevant fluorescence channels for the data shown in Figure 2d. Source data are provided as a source data file.

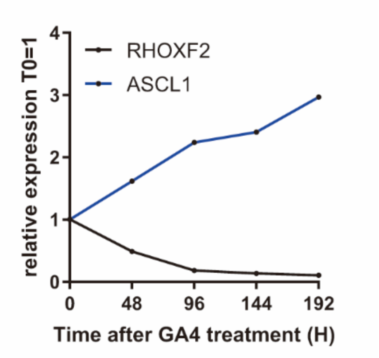

Supplementary Fig. 4. Optimization of tandem GPC circuit for sequential sgRNA expression. Kinetics of sgRNA expression and decay after excision of GPC. Tandem GPCv2-CRISPRa was treated with gibberellin for the indicated durations. RT-qPCR quantification of the relative expression level of the target genes RHOXF2, ASCL1, the target of the first and second payload sgRNA, respectively (n=2, values indicate mean± s.d.). Source data are provided as a source data file.

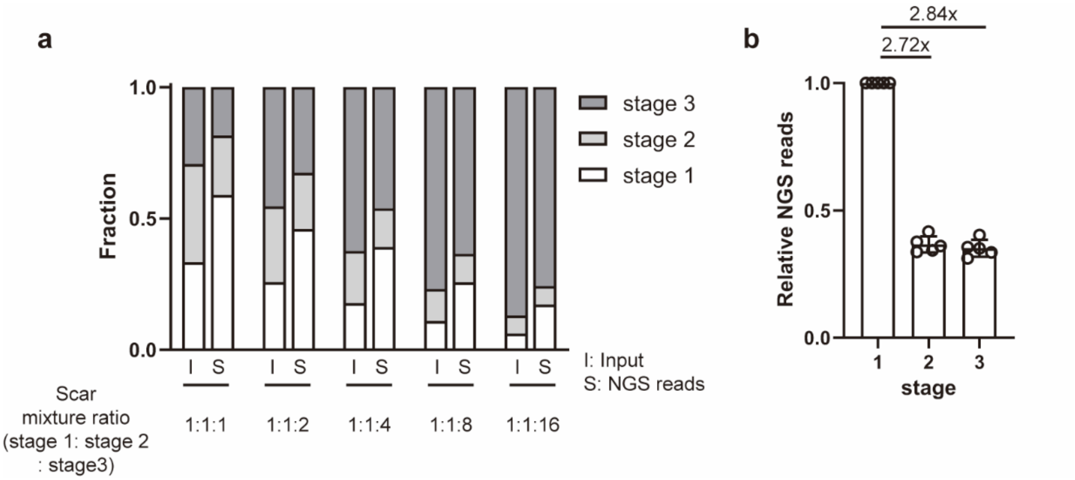

Supplementary Fig 5. PCR bias of the scar sequence amplification. a. Ratio of each scar DNA detected by NGS reads (S) compared to the ratio of each scar DNA in the input (I) DNA used for PCR amplification and subsequent NGS analysis. b. Relative sequence reads for scar sequence of each stage when they are mixed together (n=5, mean± s.d.). Source data are provided as a source data file.

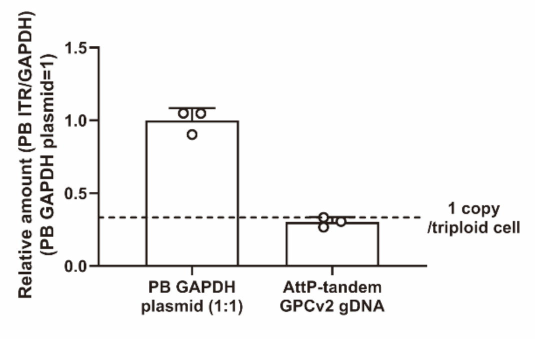

Supplementary Fig. 6. Copy number of the gene circuit in the cells carrying AttP-tandem GPCv2 used in figure 4. (n=3, mean ± s.d.). Source data are provided as a source data file.

Supplementary table 1.
List of plasmids used in this study.

Name	Description	Used in Figure
pTK1173	EFS-LoxPGIBCre251-GFP-LoxP-BFP	Supplementary Fig. 1a
pTK1174	EFS-LoxPGIBCre229-GFP-LoxP-BFP	Supplementary Fig. 1a
pTK1175	EFS-LoxPGIBCre256-GFP-LoxP-BFP	Supplementary Fig. 1a
pTK1176	EFS-LoxPGIBCre270-GFP-LoxP-BFP	Supplementary Fig. 1a
pTK1181	EFS-LoxPGIBCre229-GFP-LoxP-BFP with 4x-1xNLS	Supplementary Fig. 1b
pTK1182	EFS-LoxPGIBCre229-GFP-LoxP-BFP with 1x-4xNLS	Supplementary Fig. 1b
pTK1183	EFS-LoxPGIBCre229-GFP-LoxP-BFP with 4x-4xNLS	Supplementary Fig. 1b
pTK1184	EFS-LoxPGIBCre251-GFP-LoxP-BFP with 4x-1xNLS	Supplementary Fig. 1b
pTK1185	EFS-LoxPGIBCre251-GFP-LoxP-BFP with 1x-4xNLS	Supplementary Fig. 1b
pTK1186	EFS-LoxPGIBCre251-GFP-LoxP-BFP with 4x-4xNLS	Supplementary Fig. 1b
pTK1187	EFS-LoxPGIBCre270-GFP-LoxP-BFP with 4x-1xNLS	Supplementary Fig. 1b
pTK1188	EFS-LoxPGIBCre270-GFP-LoxP-BFP with 1x-4xNLS	Supplementary Fig. 1b
pTK1189	EFS-LoxPGIBCre270-GFP-LoxP-BFP with 4x-4xNLS	Figs. 1c-e, Supplementary Fig. 1b, Supplementary Fig. 2c
pTK1224	EFS-FRT ABAFlpO-GFP-FRT-BFP	Supplementary Fig. 1b
pTK1225	EFS-FRT ABAFlpO-GFP-FRT-BFP with 4x-1xNLS	Figs. 1c-e, Supplementary Fig. 1b, Supplementary Fig. 2c
pTK1226	EFS-FRT ABAFlpO-GFP-FRT-BFP with 1x-4xNLS	Supplementary Fig. 1b
pTK1227	EFS-FRT ABAFlpO-GFP-FRT-BFP with 4x-4xNLS	Supplementary Fig. 1b
pTK1196	EFS AttP-GIBPhiC233-GFP-AttB-BFP	Supplementary Fig. 1a
pTK1197	EFS AttP-GIBPhiC396-GFP-AttB-BFP	Supplementary Fig. 1a
pTK1198	EFS AttP-GIBPhiC428-GFP-AttB-BFP	Supplementary Fig. 1a

pTK1199	EFS AttP-GIBPhiC571-GFP-AttB-BFP	Supplementary Fig. 1a
pTK1204	EFS AttP-GIBPhiC233-GFP-AttB-BFP with 4x-1xNLS	Supplementary Fig. 1b
pTK1205	EFS AttP-GIBPhiC233-GFP-AttB-BFP with 1x-4xNLS	Supplementary Fig. 1b
pTK1206	EFS AttP-GIBPhiC233-GFP-AttB-BFP with 4x-4xNLS	Figs. 1c-e, Supplementary Fig. 1b
pTK1423	EFS-LoxP-GIBCre270 Y324F GFP-LoxP-BFP with 4x-4xNLS	Figs. 1c-e
pTK1115	PB CAG-3xSVpA-mCherry	Supplementary Fig. 1c
pTK1116	PB CAG-1xSVpA-mCherry	Supplementary Fig. 1c
pTK1117	PB CAG-BGHpA-mCherry	Supplementary Fig. 1c
pTK1118	PB CAG-rbGlobpA-mCherry	Supplementary Fig. 1c
pTK1119	PB CAG-HSVpA-mCherry	Supplementary Fig. 1c
pTK1230	PB CAG-2xSapI-iRFP w/o PEST for tGPC v1	Supplementary Fig. 2e
pTK1263	PB CAG-iRFP720 w/o PEST for tGPC v2	Figs. 2b-e, Supplementary Fig. 2a, Supplementary Figs. 2e-f
pTK1442	EFS-LoxPGIBCre270-ZeoBFP-3xBGH-2xcHS4 with 4x-4xNLS for tGPC v1	Supplementary Fig. 2e
pTK1443	EFS-FRT-ABAFlpO-GFP-3xBGH-2xcHS4 with 1x-4xNLS for tGPC v1	Supplementary Fig. 2e
pTK1444	EFS-AttP-GIBCphiC-mCherry-3xBGH-2xcHS4 with 4x-4xNLS for tGPC v1	Supplementary Fig. 2e
pTK1445	EFS-LoxPGIBCre270-ZeoBFP-3xBGH-2xcHS4 with 4x-4xNLS for tGPC v2	Figs. 2b-e, Supplementary Fig. 2a, Supplementary Figs. 2e-f, Supplementary Fig. 3
pTK1446	EFS-FRT-ABAFlpO-GFP-3xBGH-2xcHS4 with 1x-4xNLS for tGPC v2	Figs. 2b-e, Supplementary Fig. 2a, Supplementary Figs. 2e-f, Supplementary Fig. 3
pTK1447	EFS-AttP-GIBCphiC-mCherry-3xBGH-2xcHS4 with 4x-4xNLS for tGPC v2	Figs. 2b-e, Supplementary Fig. 2a, Supplementary Figs. 2e-f,

		Supplementary Fig. 3
pTK1312	PB EF1a-dCas9-VPR-P2a-Csy4-CAG-28-sgTTN-28	Fig. 3a
pTK1313	PB EF1a-dCas9-VPR-P2a-CAG-tRNA$^{Gly\ C55G}$-sgTTN-tRNA^{Gly}	Fig. 3a
pTK1314	PB EF1a-dCas9-VPR-P2a-Csy4-CAG-20-sgTTN-20	Fig. 3a
pTK1328	PB EF1a-dCas9-VPR-P2a-CAG-HH-sgTTN-HDV	Fig. 3a
pTK1280	FUW sgTTN-CMV$_{minP}$-RFP-CMV-Puro	Fig. 3a
pTK1448	EFS-LoxPGIBCre270-Zeo-20-sgRHOXF2-20-3xBGHpA-2xcHS4 with 4x-4xNLS	Figs. 3b-c, Supplementary Fig. 3
pTK1449	EFS-FRT-ABAFlpO-20-sgASCL1-20-3xBGHpA-2xcHS4 with 1x-4xNLS	Figs. 3b-c, Supplementary Fig. 4, 3b-c
pTK1450	EFS-AttP-GIBCphiC-20-sgHBG-20-3xBGHpA-2xcHS4 with 4x-4xNLS	Figs. 3b-c, Supplementary Fig. 4
pTK1451	EFS-LoxPGIBCre270-Zeo-20-sgAPC-20-3xBGHpA-2xcHS4 with 4x-4xNLS	Figs. 3d-g, Figs. 4a-f
pTK1452	EFS-FRT-ABAFlpO-20-sgMLH1-20-3xBGHpA-2xcHS4 with 1x-4xNLS	Figs. 3d-g, Figs. 4a-f
pTK1453	EFS-AttP-GIBphiC-20-sgSMAD4-20-3xBGHpA-2xcHS4 with 4x-4xNLS	Figs. 3d-g
pTK1492	EFS-GIBphiC-20-Puro-sgSMAD4-20-3xBGHpA-2xcHS4 with 4x-4xNLS w/o AttP	Figs. 4a-f
pTK1501	PB CAG-Cas9-P2a-Csy4-P2a-2xSapI-sgTP53	Figs. 3d-g
pTK1502	PB CAG-dCas9-VPR-P2a-Csy4-P2a-2xSapI-sgTTN	Figs. 3b-c, Supplementary Fig. 4
pTK1503	PB AttP-CAG-Cas9-P2a-Csy4-P2a-2xSapI	Figs. 4a-f
pTK1601	tGPC Fluor v1=pTK1230+1442+1443+1444	Supplementary Fig. 2e
pTK1602	tGPC Fluor v2=pTK1263+1445+1446+1447	Figs. 2b-e, Supplementary Fig. 2a, Supplementary Figs. 2e-f, Supplementary Fig. 3
pTK1603	tGPC-CRISPRa=pTK1502+1448+1449+1450	Figs. 3b-c, Supplementary Fig. 4
pTK1604	tGPC-CRISPR=pTK1501+1451+1452+1453	Figs. 3d-g
pTK1605	tGPC-AttP-CRISPR=pTK1503+1451+1452+1492	Figs. 4a-f

Supplementary table 2.

Protospacer sequences of sgRNA used in this study.

Gene	Sequence
RHOXF2	AACGCGTGCTCTCCCTCATC
ASCL1	CGGGAGAAAGGAACGGGAGG
HBG	CTTGACCAATAGCCTTGACA
TTN	CCTTGGTGAAGTCTCCTTTG
APC	TCTGTATAAATGGCTCATCG
MLH1	TAATAGTAACATGAGCCACA
SMAD4	TGTCCTTCAGTGGACAACGA
TP53	CCATTGTTCAATATCGTCCG

Supplementary table 3.

PCR primers for RT-qPCR.

Gene	Sequence
RHOXF2	F: GGCAAGAAGCATGAATGTGA
	R: GCCATTAATGCCCTCTGATG
ASCL1	F: CGCGGCCAACAAGAAGATG
	R: CGACGAGTAGGATGAGACCG
HBG	F: AGATGCCACAAAGCACCTG
	R: CTGCAGTCACCATCTTCTGC
TTN	F: TGTTGCCACTGGTGCTAAAG
	R: ACAGCAGTCTTCTCCGCTTC

Supplementary table 4.

PCR primers used for T7 endonuclease assay.

Gene	Sequence
APC	F: CCCTAGAACCAAATCCAGCA
	R: TATCATCCCCCGGTGTAAAA
MLH1	F: GACCCCGTCATAGCACAGTT
	R: GCAAAGGGGCAGAAATTACA
SMAD4	F: TTGGGTTGGGTTTCTAGAGG
	R: GTGGAAGCCACAGGAATGTT
TP53	F: AGGGTGTGATGGGATGGATA
	R: CTGCCCTGGTAGGTTTTCTG

Supplementary table 5.

PCR primers used for next generation sequencing analysis to quantify indel frequency. Six-nucleotide barcodes "NNNNNN" were used for multiplexing.

Gene	Sequence
APC	F: NNNNNNTCCAGGTTCTTCCAGATGCT
	R: TTTTTCTGCCTCTTTCTCTTGG
MLH1	F: NNNNNNCCCTTTGGTGAGGTGACAGT
	R: CGTACTCAAGATCTCTGCCAAA
SMAD4	F: NNNNNNTCAAGTATGATGGTGAAGGATGA
	R: CTTGTGGAAGCCACAGGAAT
TP53	F: NNNNNNCTGCCCTGGTAGGTTTTCTG
	R: CCTGGTCCTCTGACTGCTCT

Supplementary Table 6
Primers used for quantitative PCR analysis of the gene circuit excision efficiency.

Gene	Sequence
Piggybac 5’ ITR	F: GCTGCTGTGCATTTAGGACA
	R: CACGCGGTCGTTATAGTTCA
Cas9	F: CCGAAGAGGTCGTGAAGAAG
	R: GCCTTATCCAGTTCGCTCAG
GAPDH (genomic)	F: CGTAGCTCAGGCCTCAAGAC
	R: GTCGAACAGGAGGAGCAGAG
