# Supplementary material for: Scalable recombinase-based gene expression cascades
Source: Nat Commun. 2021 May 11;12:2711. doi: 10.1038/s41467-021-22978-4 (PMC8113245; doi:10.1038/s41467-021-22978-4)
Supplement: Supplementary file 2 — Reporting Summary [file 41467_2021_22978_MOESM2_ESM.pdf]

nature research

Corresponding author(s): *Double-blind peer review submissions: write DBPR and your manuscript number here instead of author names.*

Last updated by author(s): Nov 6, 2020

Reporting Summary

Nature Research wishes to improve the reproducibility of the work that we publish. This form provides structure for consistency and transparency in reporting. For further information on Nature Research policies, see our Editorial Policies and the Editorial Policy Checklist.

Statistics

For all statistical analyses, confirm that the following items are present in the figure legend, table legend, main text, or Methods section.

n/a	Confirmed	
☐	☒	The exact sample size (n) for each experimental group/condition, given as a discrete number and unit of measurement
☐	☒	A statement on whether measurements were taken from distinct samples or whether the same sample was measured repeatedly
☐	☒	The statistical test(s) used AND whether they are one- or two-sided<br>*Only common tests should be described solely by name; describe more complex techniques in the Methods section.*
☐	☒	A description of all covariates tested
☐	☒	A description of any assumptions or corrections, such as tests of normality and adjustment for multiple comparisons
☐	☒	A full description of the statistical parameters including central tendency (e.g. means) or other basic estimates (e.g. regression coefficient) AND variation (e.g. standard deviation) or associated estimates of uncertainty (e.g. confidence intervals)
☐	☒	For null hypothesis testing, the test statistic (e.g. F, t, r) with confidence intervals, effect sizes, degrees of freedom and P value noted<br>*Give P values as exact values whenever suitable.*
☐	☒	For Bayesian analysis, information on the choice of priors and Markov chain Monte Carlo settings
☐	☒	For hierarchical and complex designs, identification of the appropriate level for tests and full reporting of outcomes
☒	☐	Estimates of effect sizes (e.g. Cohen's d, Pearson's r), indicating how they were calculated

Our web collection on statistics for biologists contains articles on many of the points above.

Software and code

Policy information about availability of computer code

Data collection	no software was used.
Data analysis	Custom code was used to demultiplex Next Generation Sequencing data and quantify scar sequences. The code is available ing github repository (https://github.com/tackhoonkim/GPC-NatComms2021). The indel frequency was quantified with CRISPRESSO2 (Clement, Nat. Biotech. 2019). The flow cytometry data were analyzed with FlowJo version 10.

For manuscripts utilizing custom algorithms or software that are central to the research but not yet described in published literature, software must be made available to editors and reviewers. We strongly encourage code deposition in a community repository (e.g. GitHub). See the Nature Research guidelines for submitting code & software for further information.

Data

Policy information about availability of data

All manuscripts must include a data availability statement. This statement should provide the following information, where applicable:

- Accession codes, unique identifiers, or web links for publicly available datasets
- A list of figures that have associated raw data
- A description of any restrictions on data availability

-Next generation sequencing data are deposited in NCBI SRA PRJNA680170: SRR13106981, SRR13106982.
-Figs. 1d, 2c, 2d, 3a, 3c, 3f, 4b, 4d-f, Supplementary Figs. 1a-c, Supplementary Figs. 2a-b, Supplementary Figs. 2e-f, Supplementary Fig. 3b, Supplementary Fig. 4, supplementary Figs. 5a-b, supplementary Fig. 6 have associated raw data.
-There are no restrictions on data availability.

Field-specific reporting

Please select the one below that is the best fit for your research. If you are not sure, read the appropriate sections before making your selection.

- [x] Life sciences
- [ ] Behavioural & social sciences
- [ ] Ecological, evolutionary & environmental sciences

For a reference copy of the document with all sections, see nature.com/documents/nr-reporting-summary-flat.pdf

Life sciences study design

All studies must disclose on these points even when the disclosure is negative.

Sample size	Sample sizes were 2 or 3 for most experiments as per the standards of the field (e.g. Chavez, Nat. Methods, 2015).
Data exclusions	No data are excluded.
Replication	Data were analyzed over multiple biological replicates. The results were reproducible over at least two independent experiments.
Randomization	HEK293T cells and their derivatives were used. As we performed experiments with a cell line with defined genetic background, there were no need for randomization.
Blinding	The investigators were not blinded. As we performed experiments with a cell line with defined genetic background, there were no need for blinding.

Reporting for specific materials, systems and methods

We require information from authors about some types of materials, experimental systems and methods used in many studies. Here, indicate whether each material, system or method listed is relevant to your study. If you are not sure if a list item applies to your research, read the appropriate section before selecting a response.

Materials & experimental systems

n/a	Involved in the study	
[x]	[ ]	Antibodies
[ ]	[x]	Eukaryotic cell lines
[x]	[ ]	Palaeontology and archaeology
[x]	[ ]	Animals and other organisms
[x]	[ ]	Human research participants
[x]	[ ]	Clinical data
[x]	[ ]	Dual use research of concern

Methods

n/a	Involved in the study	
[x]	[ ]	ChIP-seq
[ ]	[x]	Flow cytometry
[x]	[ ]	MRI-based neuroimaging

Eukaryotic cell lines

Policy information about cell lines

Cell line source(s)	HEK293T: American Type Culture Collection
Authentication	HEK293T Cells are authenticated by ATCC by short tandem repeat profiling.
Mycoplasma contamination	Cells are tested negative for Mycoplasma contamination
Commonly misidentified lines (See ICLAC register)	None of the commonly misidentified cell lines are used for this study.

Flow Cytometry

Plots

Confirm that:

- [x] The axis labels state the marker and fluorochrome used (e.g. CD4-FITC).
- [x] The axis scales are clearly visible. Include numbers along axes only for bottom left plot of group (a 'group' is an analysis of identical markers).
- [x] All plots are contour plots with outliers or pseudocolor plots.
- [x] A numerical value for number of cells or percentage (with statistics) is provided.

Methodology

Sample preparation	HEK293T cells were trypsinized and resuspended in DMEM supplemented with 10% FBS.
Instrument	BD LSR Fortessa
Software	Flowjo v.10.
Cell population abundance	Not applicable. Cells were never sorted in this study.
Gating strategy	FSC/SSC, FSC-A/FSC-H polygon gates were applied to specify living singlet cells. Polygon gates for detecting fluorescent proteins were drawn not to include untransfected HEK293T cells.

☒ Tick this box to confirm that a figure exemplifying the gating strategy is provided in the Supplementary Information.
